# Will guidelines on alcohol consumption be personalized by a genetic approach?

**DOI:** 10.1186/s12263-021-00682-8

**Published:** 2021-01-25

**Authors:** Simona Costanzo, Fabio Virgili, Salvatore Panico

**Affiliations:** 1 Department of Epidemiology and Prevention, IRCCS Neuromed, Pozzilli, IS Italy; 2 Council for Agricultural Research and Economics, Research Centre for Food and Nutrition, Rome, Italy; 3 Dipartimento di Medicina Clinica e Chirurgia, Federico II University, Naples, Italy

**Keywords:** Alcohol consumption, Genetic susceptibility, Epidemiology

Articles in this series are in the format of an interview, where the “interviewer” is a scientist who proposes the topic to be discussed, chooses the two “discussants” and prepares few specific questions to be debated, somehow in agreement with them, and finally, assembles the article on the basis of their answers.

In this specific case, the “interviewer” is the Editor of BMC *Genes and Nutrition*.

The theme of this face-to-face article stems from the recent “shift” of several nutritional guidelines from the previous suggested/recommended low-to-moderate alcohol consumption to advised against at any level, a shift being mainly driven by epidemiological observations on alcohol intake-cancer association. This topic appears to be still controversial in particular as related to the prevention of the harmful effects of alcohol consumption, possibly due to genetic susceptibility. In order to clear this open issue, we have invited two outstanding scientists to defend their position:

Simona Costanzo, PhD, is a Senior Investigator, as an epidemiologist, at the Department of Epidemiology and Prevention, IRCCS Neuromed, Italy. She performed several meta-analyses of observational studies to investigate the effect of alcohol intake at different doses on total mortality and on fatal and non-fatal cardiovascular events, showing that regular and low alcohol consumption is associated with a reduced cardiovascular and total mortality risk, whereas larger amounts with an increased risk. She is presently acting as the data manager of the Moli-sani Study (a large prospective cohort study on nutrition/genetic interactions in the risk of CVD, cancer, and neurological diseases), CORIST Collaboration (a retrospective observational study on hospitalized COVID-19 patients), and several other ongoing epidemiological studies.

Salvatore Panico, MD, MSc (LSHTM), is Professor of Internal Medicine at Federico II University of Naples, Italy. A clinician and epidemiologist, he acts as investigator in several population studies on chronic disease etiology (cardiovascular and cancer). Among them are Progetto CUORE—Epidemiology and Prevention of cardiovascular disease in Italy and EPIC Study (European Prospective Investigation into Cancer and Nutrition). In both studies, he is one of the Principal Investigators. He was designated as a member of the Scientific National Committee of the Italian Centre for Disease Control (2004–2008).

## The debate

*Question 1. The majority of efforts in evaluating genetic determinants in the associations between alcohol consumption and health have been done as concerning genotypes predisposing to addiction. None is available, so far, dealing with long-term consequences, either bad or good, on chronic diseases. Do you believe that available databases, either resulting from GWAS or biobanks (such as UKbiobank) can provide some background to this issue?*

Panico: When dealing with alcohol consumption in societies, it is important to separate alcoholism from habitual “cultural” consumption of alcohol. Nevertheless, it is also important to define a border line that limits a “safe” consumption. A number of studies have evaluated the genetic predisposition to alcohol addiction and analyzed several mechanisms potentially influenced by genetic assets, but the overall evidence is poorly informative. Identification of possible genetic assets that may influence alcohol dependence through GWAS has not produced encouraging results [1]. Moreover, the interaction between genetic susceptibility to alcoholism and socio-economic/behavioral conditions are really difficult to be disentangled, making it difficult a clear statement on the issue. Within the framework of a habitual “cultural” consumption of alcohol (not alcoholism) information on genetics as a determinant of the effects of alcohol on diseases is not available. More information is available on the mechanisms potentially responsible of the pathogenic effect of alcohol on cancer that can act through genetic modifications: carcinogenicity of ethanol in experimental animals has been well documented and IARC has categorized alcohol as a carcinogen [2, 3]. A carcinogenic effect of chemicals other than ethanol in alcoholic beverages such as nitrosamines (facilitating the absorption of other carcinogens), an inhibition of methylation caused by ethanol, or a carcinogenic and genotoxic role for acetaldehyde, the major metabolite of ethanol, are also known. Acetaldehyde is cytotoxic, mutagenic, and carcinogenic and has been shown to be responsible for tumor-enhancing effects leading to aberrant cell proliferation and bind to DNA, leading to the formation of stable DNA adducts [4, 5]. Epigenetic alterations (such as selective acetylation, methylation, and phosphorylation of histones) have also been identified as possible pathogenic mechanism [6, 7].

The development of several large projects including information on alcohol consumption and the support of a biological specimen bank has stimulated the scientific community to develop and evaluate hypothesis that could explain the role of genetics in determining the pathogenic effects of alcohol consumption. This path could provide crucial information for a precision medicine knowledge, possibly identifying individuals with a concrete risk of disease from alcohol consumption. Most projects are based on prospective analysis of very large cohorts; a fact that may encourage valid analyses. As a component of the Steering Committee of the EPIC Study (500,000 individuals followed-up for many years to evaluate chronic disease incidence and mortality, especially cancer, and with baseline information on alcohol consumption), I have participated to many discussions to find the golden bullet hypothesis to be studied [8]. In the absence of a promising hypothesis, energy of the researchers has been spent essentially on the effects of alcohol consumption on the genetics or epigenetic assets able to produce specific cancers. EPIC has a large database on alcohol consumption including lifetime consumption, although scarce information has been collected on drinking patterns.

The collection of relevant and crucial information on alcohol consumption is fundamental; in fact, a lack of precision in the alcohol consumption in UK biobank has been claimed and may explain in part the absence of a clear effect of alcohol in analysis performed on UK biobank data (in terms of genetic influence on its consumption and detection of pathogenic effects on cancers) [9]. Apart from the need for a comprehensive information on alcohol consumption, the real question is to try to identify specific genetic driven hypotheses to be tested.

Costanzo: I agree with Dr. Panico on the importance to distinguish the well-documented harmful effect of alcohol misuse (abuse, drunkenness, binge drinking, alcoholism) from the effects on health of a regular and moderate alcohol consumption, preferably during meals.

Although there is general agreement that heavy alcohol is associated with increase of several different diseases and all-cause mortality, some public organizations insist on considering alcohol to be harmful even when consumed in light amounts (“zero tolerance”), resulting in conflicting messages through media and in different alcohol guidelines [3, 10].

The scientific community generally agrees on the existence of a J-shaped curve between alcohol consumption and all-cause mortality. The accepted interpretation of the J-shaped curve relating alcohol intake to mortality is that the lowest point on the curve (light-to-moderate drinking, one to two drinks per day) represents optimum exposure to alcohol, while the increased risk in non-drinkers or heavy drinkers reflects the consequence of sub-optimal exposure [10, 11].

In recent years, epidemiologists have increasingly sought to employ genetic data to identify “causal” relationships between exposures of interest and various endpoints, by utilizing genetic variants that are reliably associated with the potentially modifiable risk factor under investigation. However, this approach is subject to all limitations of instrumental variable analysis and to several limitations specific to its genetic underpinnings, including confounding, weak instrument bias, pleiotropy, adaptation, and failure of replication [12].

Recently, several studies have used Mendelian randomization as a new approach to investigate the association between alcohol consumption and health [9]. In general, these observational studies using genetic variants as proxies or instruments for alcohol consumption resulted into inconsistent conclusions.

The Mendelian randomization approach applied to alcohol epidemiology is questionable for two main reasons.

Indeed, when the genetic regulation of a phenotype is strong, stable over time, and marginally influenced by non-genetic (environmental) factors, the Mendelian randomization approach is more appropriate. But in the case of alcohol, Mendelian randomization investigates the association between a “genetic predisposition” to consume alcohol (at any dose) and the outcome. The polymorphisms that “regulate” its consumption actually have limited impact on the phenotype, which is on the contrary largely influenced by environmental/cultural factors. Second, the crucial issue is whether drinking in moderation, say a drink a day, is better for the health than not drinking at all. From a Mendelian randomization perspective, this would require a targeted genetic analysis comparing light-drinkers vs abstainers, a study that has not been carried out so far, to the best of our knowledge. If we compare (at any daily dose) drinkers vs non-drinkers, it may well happen that identified-by-polymorphisms drinkers (without any reliable distinction on consumption levels) are at higher outcome risk in comparison with identified-by-polymorphisms non-drinkers [10].

Of note, genetic analyses are also prone to their own forms of confounding when specific genetic variants tend to track with specific subpopulations (e.g., the ADH1B rs1229984 variant with individuals of East Asian and Middle-Eastern descent); this type of confounding can only be statistically addressed if genetic substructure has characterized insufficient details to identify the carriers of interest, a variable occurrence in most cohort studies [13]. This suggests that the effects of different social, environmental, and lifestyle conditions in various regions may overcome that attributable to the studied polymorphisms, greatly limiting the value of that Mendelian randomization study. Finally, according to epigenetic studies, these and other environmental factors may modify gene expression in an uncontrolled way, thus further limiting the scientific value of a Mendelian randomization.

*Question 2. According to the points above, do you think that providing personalized nutritional advices, based on the presence of specific genotypes differentially predisposing to adverse effects of alcohol consumption even at low-moderate levels, can be an expedient strategy to discriminate specific subgroups of the population?*

Panico: In spite of some data on the genetic susceptibility to the effect of alcohol consumption indicating the role of polymorphism in favoring some disease, such as for colon cancer [14], we are still unable to identify those genetic characteristics that can be used for a selection of individuals with high risk of complications of chronic and degenerative diseases associated to alcohol consumption (especially low-moderate). The kind of evidence available suggest some interactions between alcohol consumption and the genetic modification due to this consumption, concerning especially aldehyde dehydrogenase-2 (ALDH2) and alcohol dehydrogenase (ADH), but the effect size is really trivial to provide indications for a convincing trace to intervention inspired to genetic knowledge [15, 16]. Therefore, our target for prevention of damages due to alcohol is still the overall population. One should say that even when we will have more detailed information on individuals at high risk conditions, we cannot support a “break the lines” policy for the others. Alcohol is classified as a carcinogen [2, 3] and a warning on amount of consumption will be necessary for everybody. Moreover, the mechanisms of the damage from alcohol are different according to different diseases (in the case of cancer, the specific site) and it is unlikely that we could measure the overall genetic risk of a single individual. Currently on the basis of scientific evidence, we know that individuals eating a specific dietary pattern (i.e., Mediterranean) can drink alcoholic beverages more safely than others, although under specific conditions (preferably during meals) [17–22].

Costanzo: Beneficial or harmful outcomes derived from moderate or heavy alcohol consumption are a crucial issue in public health and the setting of appropriate strategies of prevention/intervention for future public health activities cannot be only based on the presence of specific genotypes.

Evidence from studies on twin, family, and adoption showed that genetics plays an important role in determining an individual’s preferences for alcohol and his or her likelihood for developing alcoholism [23]. However, alcoholism doesn’t follow the simple rules of inheritance set out by Mendel, being influenced by several genes that interact with each other and with environmental factors.

Without any doubt, genetic susceptibility to specific diseases is modifiable by the level of alcohol consumption, but we should consider that this complex exposure is strongly influenced by culture, religion, socio-economic conditions, local traditions.

*Question 3. How do you think national guidelines can be realistically followed by the population? Can you imagine the “third time” following a rugby match toasted with mineral water? Very strong cultural habits push in the opposite direction supported by specific economic reasons, in particular in specific countries (Italy, France, USA) where wine production has a significant impact in overall economy. This attitude is further corroborated by a plethora of “popular”, though scientifically robust, publications reporting alcohol drinking (usually wine) as an expedient “anti-aging”, health-protecting strategy. Why should we not believe it?*

Panico: The issue is: do we need to rely on scientific evidence or the habitual “culture” is more important? Scientific evidence indicates that the frequent use of amounts of alcoholic beverages higher than suggested may be dangerous for health [24]. As soon as much evidence has been accumulated, we have realized that a number of “popular” information has to be considered “popular beliefs”. Some questions have still to be solved, i.e., is a slightly higher amount of frequent use of alcohol beverages (wine or beer) still be safe when consumed during “protective meals” like those inspired to Mediterranean style? Looking at literature, the extra amount is a second glass of wine or an equivalent amount (in terms of alcohol) of beer [17–19]. It is reasonable to say that the most important obstacle to a different culture on alcohol consumption is the pressure of the alcoholic beverages producers; this is a relevant issue for countries like Italy, France, and USA. We need to realize that this issue is a piece of a puzzle that, as human beings, we have to face in the future (close and remote): we need to reduce the consumption of any kind of goods (earth resources are limited), and implement items that are safe for our health. The implementation of educational programs to learn how to reduce all the consumptions may help to understand that in the everyday life we need to have essential consumptions, limiting at specific occasions a number of habits including dietary ones. Within this framework the “third time” following a rugby match is not a problem provided that alcohol beverages exceeding the suggested amount are consumed only in that limited occasion and avoiding very high amount.

Costanzo: Actually, national guidelines in various frameworks, and specifically in the context of nutrition, are usually poorly observed by the population. Cultural pressure can be more efficacious than scientific guidelines. As an example, in Italy, the concept that alcohol in moderation during meals is a healthy habit is largely recognized in popular culture since a long time (we can of course discuss about a precise definition of “moderate”, taking into account that alcoholic beverages are not drugs; for the latter only, precise quantitative amounts are defined and prescribed). However, this solid and diffuse popular cognition failed up to now to avoid the quite recent increase in binge drinking habits among the younger population. Binge drinking in special occasions (parties, weekends) is an Anglo-Saxon practice that in the recent years spread out in Italy, despite both restrictive national guidelines and “ancient popular culture”.

*Question 4. Is the genetic profile associated to metabolic tolerance the solely reason in determining alcohol addiction? Do we have any indication of other variants associated to taste perception or reward mechanisms?*

Panico: As pointed out before, data on genetic influence on alcohol addiction or habitual “cultural” alcohol consumption are not informative to allow possible actions to prevent (or cure) alcoholism and modulate the unfavorable effect of alcohol on diseases. As to habitual “cultural” alcohol consumption, we know that taste perception may influence drinking habits or patterns: some individuals prefer light wines instead of hard tasting ones; specific flavor in wines may discriminate wine buyers; some individuals confine the consumption of alcoholic beverages during meals. Following this knowledge, there are attempts to implement bitterness or burning taste to structure a sensory hindrance to alcohol overconsumption [25]. Certainly, the psychological reward mechanisms are important to influence drinking pattern especially among younger individuals [26], but it is unreasonable to believe that they cannot be influenced by several types of interventions including a strong educational campaign to reduce excess of alcohol consumption.

Costanzo: How taste influences alcohol behavior was investigated in the last decade, because a better understanding of the factors that influence alcoholic beverage preference and consumption is important for disease prevention and management [25, 27]. The ability to taste bitterness affects food choices and alcohol consumption and in particular, ethanol is generally aversive as it primarily elicits bitterness and irritation when ingested. Individuals who experience orosensations more intensely tend to report lower liking and consumption of alcoholic beverages. Additionally, a preference for sweetness is likely associated with a paternal history of alcohol use disorders [27].

On the other hand, a recent study hypothesized that the flavor of beer constitutes could be a conditioned stimulus associated with alcohol reward. Therefore, it was investigated whether oral exposure to beer with or without alcohol elicits similar brain responses in reward related areas, in a context where drinking regular alcoholic beer is expected [28]. During tasting and swallowing, there were no significant differences between the two beers in acute brain reward, suggesting that in regular consumers, beer flavor rather than the presence of alcohol is the main driver of the consumption experience.

*Question 5. The majority of pathologies associated to alcohol consumption is likely to be significantly modulated by intestinal microbiota which, in turn, is affected by alcohol intake. Can we speculate about other nutritional variables significantly contributing to alcohol-disease relationship (binge vs. drinking wine at meals; occasional vs regular drinking; diet composition; nutrients and non-nutrient bio-actives intake)?*

Panico: Studies have shown that alcohol consumption affects the microbiota both at oral and intestinal level [29, 30]. The greatest evidence is for heavy alcohol consumption; in fact, in heavy drinkers, the information is quite clear. We know that some of the microbiota components can change toward a pattern more similar to that of non-drinkers after abstinence program [29]. The major issue with microbiota is that it is difficult to evaluate the components of the pattern of bacteria as possible mediating factors to determine diseases attributed to alcohol consumption. We can rely on indirect evidence. For example, fiber consumption modulates the effect of alcohol on breast cancer (we know that alcohol is a classic risk factor for this cancer), reducing the risk; this effect may be attributable to the effect of fibers on microbiota [31]. We can speculate that the protective effect of Mediterranean diet on unfavorable alcohol consumption is associated with a specific microbiota pattern, typical of Mediterranean eating individuals [32]. As pointed out before, types of alcohol beverages and drinking patterns may play an important role in mitigating the unfavorable effect of alcohol consumption: drinking wine at meals is certainly safer that drinking out meals; binge drinking has been shown to be particularly dangerous in youth [33].

Costanzo: Even though alcohol consumption alone may damage the microbiota balance, moderate red wine intake has been shown to have a beneficial effect on gut microbiota. A weaker association is detected with white wine; this could be in relation to a lower content of polyphenols, such as antocyanin, resveratrol, and gallic acid, in comparison with red wine [34]. In general, polyphenols present prebiotic properties and antimicrobial activities against pathogens. For instance, polyphenols-mediated induction in the proliferation of *Bacteroides* spp. leads to a reduction in the blood pressure, a lipid profile improvement, while an enrichment of Proteobacteria population determines a lowering in uric acid levels [35, 36]. Similar to red wine, moderate beer consumption too shows positive effects on gut microbiota, mainly due to catechins and epicatechins (that are found in high concentrations in beer), which are able to repress harmful species growth [37, 38].

## Common summary

To summarize the major points expressed in replying at the questions above, Dr. Costanzo and Dr. Panico would do as follows:

- We have no comprehensive knowledge to identify genetic susceptibility of individuals at high risk of disease in consuming alcohol, that is no information is presently available for a personalized approach to alcohol drinking indications.
- Our target for prevention of alcohol-related diseases is to strongly support community campaigns to reduce heavy and irregular alcohol consumption to currently suggested moderate and regular amounts.
- Given the importance of discouraging non-drinkers to start drinking regularly alcoholic beverages, in the absence of sound information on the genetic susceptibility to alcohol consumption, those who are used to drink alcoholic beverages should be advised to confine this habit during meals, possibly inspired to Mediterranean Diet or to a diet rich in vegetable products; in any case, quite moderate consumption should be the rule.
- Educational campaigns to reduce alcohol beverages consumption need to be part of a more general campaign on generalized reduction of any food (and other goods) consumption within the framework of a wide awareness of reducing the earth resources for a sustainable future.

## The interviewer final comment

I want to propose to the readers of this debate a personal take-home message: the research on the interaction between nutrition and health is far to be concluded. The complete understanding of gene-diet interaction is still far and, even though gigantic leaps ahead are being made every day, we cannot provide yet any conclusive answer. The necessary next step will necessarily involve the characterization of specific genetic profiles and identify subjects with a high polygenic risk score for a high risk of harmful consequences of alcohol intake, even at very low quantities to specifically “tailor” and target our recommendations [39].

## Data Availability

Not applicable.
